# Systematic Evaluation of sEMG Processing Pipelines for Gesture Recognition

**DOI:** 10.3390/bioengineering13091019

**Published:** 2026-09-01

**Authors:** Elsa Concha-Pérez, Jorge A. Reyes-Avendaño, Hugo G. Gonzalez-Hernandez, Maricruz Concha-Pérez

**Affiliations:** 1 School of Engineering and Sciences, Tecnologico de Monterrey, Ave. Eugenio Garza Sada 2501 Sur, Col: Tecnológico, Monterrey 64700, Nuevo León, Mexico; a01551967@tec.mx (E.C.-P.); jareyesa@tec.mx (J.A.R.-A.); 2 Instituto Tecnológico de Orizaba, Tecnológico Nacional de México, Orizaba 94320, Veracruz, Mexico; m26010123@orizaba.tecnm.mx

**Keywords:** signal processing, experimental design, wearable sensors, consistency analysis

## Abstract

Gesture recognition enables intuitive human–robot communication, where surface electromyography (sEMG) provides a minimally invasive interface for detecting motor activity. However, the lack of systematic evaluation of processing pipelines represents a critical barrier to reliable subject-independent deployment. This work presents a systematic evaluation of sEMG processing pipelines for three-gesture recognition (Neutral, Ask, Take) using a Bagged Trees classifier under Leave-One-Subject-Out (LOSO) cross-validation across ten participants. A mixed-effects ANOVA over 1872 experimental configurations revealed that the preprocessing pipeline is the dominant factor affecting classification accuracy, followed by inter-subject variability, while window size and overlap exhibit smaller but statistically significant effects. A consistency-based elbow analysis identified a compact subset of five features that reduced input dimensionality by 94.79% while improving accuracy from 68.10% to 69.86% and reducing training time by 51.62%. Bayesian hyperparameter optimization was also tested, but it did not yield statistically significant improvements over the five-feature baseline; given the limited cohort (*n* = 10), this indicates the absence of a detectable difference and points to inter-subject variability as a leading factor limiting performance rather than model configuration. These findings provide empirically grounded guidelines for designing computationally efficient sEMG-based gesture recognition systems for collaborative robotics.

## 1. Introduction

Industry 5.0 extends the objectives of industrial digitalization by placing human well-being, sustainability, and resilience at the center of technological development [1]. Within this paradigm, human–robot collaboration (HRC) systems should not only improve productivity but also support worker autonomy, safety, and physical well-being. Bussolan et al. introduced the MultiPhysio-HRC dataset, which integrates electromyography (EMG) with other physiological and behavioral modalities collected during industrial HRC scenarios to support the development of human-aware robotic systems [2]. When users and robots interact in a shared workspace, intuitive and natural communication channels must therefore be established; gestures can be captured using cameras, inertial measurement units (IMUs), force sensors, or electrophysiological signals.

Surface electromyography (sEMG) is a type of electrophysiological signal that records muscular activation in the periphery of the central nervous system, which can be used to calculate contraction intensity, fatigue, and to detect motor intention [3]. Wearable devices that utilize sEMG have gained broad interest for use in gesture recognition, movement tracking, and intelligent control applications due to their minimally invasive nature, rapid response, and flexible design, supporting wireless communication and autonomy [4].

Recent studies illustrate the growing role of sEMG interfaces in human-centered HRC. Chand et al. used sEMG to characterize localized muscle fatigue during repetitive manufacturing operations, illustrating the value of physiological sensing for supporting worker well-being [5]. Zhang et al. subsequently developed a robust EMG-based interaction interface for recognizing human motion intention in three-dimensional HRC tasks [6]. More recently, Zafar et al. demonstrated real-time EMG-based gesture recognition on edge devices using federated learning, highlighting the importance of computational efficiency and biometric-data privacy in deployable human–robot interfaces [7]. These developments demonstrate the relevance of physiological interfaces to Industry 5.0; however, their practical adoption also requires models that generalize beyond the individuals used during development.

The nature of sEMG is complex and exhibits nonlinear dynamic behavior [8]. This complexity leads to substantial variability in the recorded data, even for the same user across different trials of the same event [9]. Inter- and intra-subject variability is therefore recognized as a central challenge in sEMG applications [10]. Moreover, despite the advantages of wearable sEMG devices, they are susceptible to surrounding electrical noise sources, cable artifacts, variations in the electrode–skin interface, unwanted body movement, sweating, the physical attributes of the subjects, and interference from other physiological signals such as cardiac electrical activity. These sources of variability distort the underlying sEMG patterns associated with motor activities [11].

Movement patterns have been identified using classification models based on machine and deep learning [12], but the data must first be preprocessed to minimize noise while preserving information related to the human activity of interest. Preprocessing steps are commonly defined according to the characteristics of the data, recommendations in the literature, and the experience of the researcher. In most cases, the signal is filtered using a Butterworth band-pass filter to remove low-frequency components such as motion artifacts, baseline drift, or impedance fluctuations, as well as high-frequency components such as electrical interference and sensor noise [13]. Recent work also identifies band-pass filtering as a standard step in sEMG processing pipelines [14]. Another common procedure is rectification, which converts negative signal amplitudes into positive values so that the magnitude of muscle activation can be represented [14]. González et al. similarly incorporate rectification into their sEMG preprocessing methodology [15]. Subsequently, the signal envelope is extracted to capture the sEMG activation profile [16]. Normalization is then applied to facilitate comparisons across subjects [17].

Beyond these traditional steps, various advanced techniques have been developed for preprocessing and analyzing sEMG data, including higher-order statistics, empirical mode decomposition, independent component analysis, artificial neural networks utilized for noise removal, or wavelet analysis to eliminate Gaussian white noise [18]. However, even with a wide range of analytical tools available, the sequential arrangement of standard preprocessing steps and their direct interaction with temporal segmentation parameters are rarely evaluated systematically, leaving a critical gap in understanding how to establish a robust framework for gesture recognition.

After the preprocessing stage, to work with machine learning models, the data are frequently segmented into temporal windows of a certain length and overlap; larger window sizes generally capture greater temporal context, improving discriminability up to a saturation point, but they are more computationally expensive [19], so a balance between the amount of context and computational efficiency must be sought. From each window, sets of time-domain, frequency-domain, or time–frequency features are computed to characterize the signals and maximize the margins among the least separable patterns [20]. Nevertheless, using a large set of features increases computational complexity, and many of these features may be redundant; consequently, a feature selection technique is employed to identify subsets of the most relevant features [21].

Once relevant features have been identified, feature vectors are translated to labels or classes; classical classification models such as Linear Discriminant Analysis (LDA), Support Vector Machines (SVMs), Random Forests (RFs), or k-Nearest Neighbors (kNNs) have been widely adopted for this purpose [14]. However, ensemble classifiers achieve more accurate and robust performance using a weighted combination of several classifier models [22]. Specifically, bagged ensembles of decision trees are effective in capturing nonlinear relationships; the Bagging (Bootstrap aggregation) technique is employed as an ensemble strategy to reduce the variance of individual trees and to prevent overfitting [23].

The design of an sEMG gesture recognition system requires coordinated decisions concerning preprocessing, temporal segmentation, feature selection, and classifier configuration. A detailed synthesis of the limitations of previous studies and the resulting research gap is provided at the end of Section 2. This synthesis motivates the research questions presented below.

Q1. Among the combinatorial space of preprocessing operations (filtering, outlier rejection, envelope computation, and normalization) and temporal segmentation settings (window size and overlap), which specific setup yields the highest gesture recognition accuracy under a subject-independent evaluation scheme, and what is the relative contribution of each factor to that accuracy?
Q2. What is the minimum number of time-domain sEMG features, selected on the basis of cross-subject consistency, required to reach peak classification performance, and what computational benefit does this dimensionality reduction provide?
Q3. Does Bayesian hyperparameter optimization of the ensemble classifier yield a statistically significant improvement over a fixed baseline configuration under the same LOSO evaluation protocol?

The purpose of this article is to systematically evaluate the joint effect of preprocessing, temporal segmentation, feature selection, and hyperparameter optimization on sEMG-based gesture recognition under a subject-independent evaluation scheme, in order to provide grounded answers to the proposed research questions and to identify a computationally efficient pipeline suitable for real-world human–robot collaboration scenarios. To this end, a factorial experimental design is combined with a mixed-effects analysis of variance (ANOVA) to determine the optimized pipeline when working with a Bagged Trees model trained and tested using a LOSO cross-validation protocol across ten participants, enabling the isolation of each factor’s contribution to classification performance. The main contributions of this work are articulated at several levels:

1. Systematic factorial evaluation of sEMG preprocessing pipelines. A mixed-effects ANOVA framework is applied to 1872 experimental configurations (16 preprocessing pipelines × 13 window sizes × 9 overlap levels) quantifying the independent and interactive effects of each factor. This offers design recommendations, supported by empirical evidence and statistical validation, for practitioners deploying gesture recognition systems using a commercial wearable sEMG device.
2. Consistency-based feature selection with elbow analysis. A cross-subject consistency metric derived from ReliefF rankings across LOSO folds is proposed as a feature selection criterion, enabling the identification of a minimal 5-feature subset that reduces the input dimensionality by 94.79% relative to the full feature set while simultaneously improving classification accuracy from 68.10% to 69.86% and reducing training time by over 50%.
3. Empirical evaluation of Bayesian hyperparameter optimization under LOSO. Five feature–hyperparameter configurations are compared under a nested Bayesian optimization scheme, demonstrating that the simple baseline with the compact feature set shows no statistically significant difference from all optimized alternatives. This finding indicates that no improvement was detected under the tested conditions, and suggests that inter-subject variability is a leading factor limiting performance, so that complex optimization cycles may not be justified when the feature representation is already compact and consistent.
4. Practical protocol for gesture-based human–robot collaboration communication. A reproducible experimental protocol and data collection workflow are described for three task-relevant gestures (Neutral, Ask, Take) in an assembly scenario, offering a validated foundation for future work on real-time and subject-independent gesture interfaces in collaborative robotic environments.

The paper is organized as follows. Section 2 reviews relevant literature on sEMG preprocessing, feature extraction, and gesture classification. The methodology is detailed in Section 3, which describes the technological resources used for the acquisition and analysis of sEMG, the experimental protocol for data collection, the data processing pipeline, and the statistical analysis methods. Section 4 presents the results of ANOVA, feature selection analysis, and hyperparameter optimization. Section 5 presents the discussion of the results, limitations, and future work. Finally, conclusions are outlined in Section 6.

## 2. Literature Review

Surface electromyography (sEMG) is widely used in human–robot collaboration (HRC), human–machine interfaces (HMI), prosthetics, and assistive robotics because it provides a direct and non-invasive channel for decoding motor commands. Guo et al. showed that sEMG electrodes can be integrated into wireless wearable systems, improving portability in collaborative environments [24]. Jie et al. emphasized that surface electrodes acquire muscular activity without injuring the user, which supports repeated and long-term interaction [25].

sEMG activity can precede visible movement by approximately 30–150 ms, providing an early indication of motor intention [26]. Nguyen showed that signal intensity, timing, and activation patterns can be represented by lightweight time-domain descriptors suitable for real-time control [27]. The literature covers individual finger flexion and extension [28], complex multi-finger gestures [29], static hand poses [30], grasp types [31], wrist motions [32], and functional activities of daily living [33]. These different gesture taxonomies, however, produce classification problems of substantially different complexity and prevent accuracy values from being compared without considering the task and evaluation protocol.

Because sEMG is nonlinear and susceptible to baseline drift, motion artifacts, physiological cross-talk, and power-line interference, preprocessing is required before feature extraction or end-to-end classification. Nguyen implemented band-pass and notch filtering followed by overlapping segmentation in a computationally lightweight embedded pipeline [27]. In contrast, Montazerin et al. processed high-density sEMG as spatial images and evaluated window lengths from 31.25 to 250 ms for transformer-based recognition [34]. These examples show that preprocessing choices depend strongly on sampling rate, sensor density, and model input. In particular, an upper cutoff of 450–500 Hz, used with acquisition systems operating above 1 kHz, cannot be transferred to a consumer wearable sampled at 500 Hz because its Nyquist frequency is 250 Hz. Therefore, cutoff frequencies should be selected from the observable spectrum and verified through the power spectral density rather than adopted solely from precedent.

Rectification and envelope extraction remain useful when the objective is to estimate muscle activation or movement onset. Esposito et al. used rectified EMG to characterize activation-related information [35], while Carvalho et al. described RMS- and low-pass-filter-based envelope estimation procedures [36]. Gesture recognition studies do not follow a single convention. Kang et al. characterized raw sEMG through handcrafted descriptors [37], whereas Mendes et al. learned discriminative representations directly from raw sequential inputs [38]. Nguyen retained filtered signals and explicit time-domain features to reduce computation [27]. Thus, raw, rectified, enveloped, and filtered representations should be treated as alternative pipeline factors rather than universally interchangeable preprocessing choices.

Temporal segmentation governs the trade-off between information content, classification stability, and response latency. Rani et al. described sliding windows as the basic mechanism for transforming a continuous sEMG stream into samples for feature extraction and classification [39]. Gopal et al. used a 200 ms window in a systematic comparison of machine and deep learning models for assistive-robot gesture recognition [40]. Montazerin et al. demonstrated that high-density spatial information can support recognition with windows as short as 31.25 ms, although their system used 128 electrodes and a specialized transformer architecture [34]. Zhang et al. found that a dual-stream transformer remained compatible with real-time recognition, but its improved accuracy incurred an average inference-time increase of 5.25 ms relative to a single-stream transformer [41]. Consequently, the frequently used 200–300 ms range is a practical starting point, not a device-independent optimum; window size and overlap must be evaluated jointly for the target sampling rate and computational platform.

Handcrafted features remain important because they offer transparent and computationally inexpensive baselines. Nguyen extracted mean absolute value (MAV), zero crossings (ZC), waveform length (WL), slope sign changes (SSC), and variance (VAR), reporting that these descriptors differentiated the target gestures while preserving low latency in an embedded robotic-hand application [27]. Jie et al. began with 31 features from 16 sEMG channels and used a two-stage particle swarm optimization procedure to reduce both features and channels [25]. Their optimized subsets, evaluated with weighted k-NN variants, outperformed subsets produced using genetic algorithms, ant-colony optimization, and principal component analysis; mean frequency and maximum fractal length were also selected consistently across subjects. However, their training and testing were performed across repeated trials within each subject, so the reported feature stability does not establish calibration-free cross-subject generalization.

Deep architectures replace explicit feature design with learned spatial and temporal representations. Mendes et al. used convolutional processing to learn spatial patterns from sEMG inputs [38]. Yang et al. used a multi-stream residual architecture to model dynamic gestures from raw sequences [42]. Karnam et al. combined CNN and bidirectional LSTM components in EMGHandNet and evaluated the architecture across five benchmark sEMG datasets, showing the benefit of jointly modeling inter-channel and temporal dependencies [43]. Montazerin et al. evaluated a compact transformer using 128-channel recordings from 20 subjects performing 65 gestures and showed that attention can simultaneously learn temporal and spatial information without handcrafted features [34]. Zhang et al. reported accuracies of 81.47%, 88.24%, and 98.95% on Ninapro DB2 Exercise B, Ninapro DB5 Exercise C, and CapgMyo DB-C, respectively; their dual-stream LST-EMG-Net improved average accuracy by 9.5% over the single-stream transformer while remaining suitable for real-time inference [41]. These findings demonstrate the capacity of deep models, but they arise from different datasets, sensor configurations, gesture sets, and validation partitions.

Traditional machine learning classifiers remain relevant when latency, interpretability, and computational cost are priorities. Montazerin et al. noted that SVMs and LDA can perform effectively on small datasets but depend on manually engineered features [34]. Jie et al. showed that feature–classifier compatibility matters: their weighted k-NN variants benefited substantially from the PSO-selected feature and channel subsets [25]. Peng et al. extracted eight time-domain features from four upper-limb motions, selected a compact subset, and found that a random-forest model maintained strong performance in both offline testing and an initial real-time validation [44]. These results support the use of simple classifiers as deployment-oriented baselines. Nevertheless, a high result obtained after subject-specific training, calibration, or random sample partitioning cannot be interpreted as evidence of performance on unseen subjects.

A structured comparison of the principal findings and evaluation implications of representative sEMG and machine learning studies is provided in Table 1.

The quantitative comparison shows that reported performance is strongly conditioned by hardware and evaluation design. For example, Montazerin et al. reported a 5.75-percentage-point increase when moving from 32 electrodes and 31.25 ms windows to 128 electrodes and 250 ms windows [34]. Jie et al. obtained an improvement of approximately 4 percentage points by replacing conventional k-NN with weighted variants [25]. Calibration-assisted LOSO produced accuracies of 87.03% and 94.53% in the datasets evaluated by Lin et al. [45], whereas the calibration-free protocol of Yang et al. yielded 89.4% and 86.9% on NinaPro DB2 and CapgMyo DBa, respectively [46]. In the present study, reducing the representation from 96 to five features increased accuracy from 68.10% to 69.86% and reduced training time by 51.62%. Nevertheless, differences in participants, gesture sets, sensor density, preprocessing, and validation protocols prevent these percentages from being interpreted as a direct ranking of the methods.

Leave-One-Subject-Out (LOSO) cross-validation is widely used to evaluate cross-subject generalization in EMG-based recognition. Yang et al. defines LOSO as an evaluation in which one participant is excluded from model development and used exclusively for testing, thereby estimating performance for a previously unseen user [47]. Lin et al. applied LOSO to inter-subject sEMG gesture recognition and showed that target-domain normalization can improve performance, although their method requires one calibration cycle from the new user [45]. More recently, Yang et al. evaluated calibration-free sEMG recognition by excluding the held-out participant from optimization and dividing the remaining participants into training and validation sets [46]. These studies demonstrate that the use of LOSO alone does not fully characterize the evaluation protocol; any normalization, feature selection, hyperparameter tuning, or adaptation involving the target participant must also be reported explicitly.

Taken together, the literature supports three robust conclusions. First, preprocessing and segmentation choices are inseparable from the sensor bandwidth and latency requirements. Second, deep models can learn rich spatiotemporal representations, whereas handcrafted features and classical classifiers remain competitive when computational efficiency is essential. Third, reported accuracy is conditional on the evaluation design: within-subject, calibrated, transfer-learning, and subject-independent protocols answer different questions. Therefore, comparisons based only on accuracy percentages can be misleading, and subject-independent claims require an explicit protocol such as LOSO in which every test subject remains excluded from model development.

Although recent studies have reported substantial progress in individual components of the sEMG processing pipeline, four specific research gaps remain when these components are considered together:

1. Preprocessing operations are commonly selected on the basis of historical precedent or heuristic criteria, while alternative processing sequences are rarely compared within a common experimental design.
2. Segmentation parameters, particularly window size and overlap, are frequently fixed at conventional values without systematic verification for consumer-grade wearable devices operating at comparatively low sampling rates.
3. Feature-selection procedures are often evaluated without quantifying whether the selected descriptors remain consistent across different held-out subjects.
4. Ensemble-classifier hyperparameter optimization is seldom compared with a fixed baseline using identical subject-independent partitions, making it unclear whether its additional computational cost produces a statistically detectable performance benefit.

Taken together, these limitations reveal a broader research gap: the absence of a unified empirical framework that evaluates preprocessing and temporal segmentation alternatives under a common cross-subject protocol and then examines the additional contributions of cross-subject-consistent feature selection and hyperparameter optimization. The present study addresses this gap by systematically screening 16 preprocessing sequences and multiple segmentation settings under LOSO cross-validation, quantifying their effects through mixed-effects analysis, evaluating the consistency of ReliefF rankings across folds, and comparing fixed and Bayesian-optimized Bagged Trees configurations.

Unlike many state-of-the-art studies that primarily propose a new classification architecture or evaluate a fixed preprocessing configuration, the present work focuses on the systematic evaluation of the processing pipeline as an integrated experimental framework. The Bagged Trees classifier is maintained as a common baseline while 1872 combinations of preprocessing sequence, window size, and overlap are evaluated under the same LOSO protocol. Their effects are quantified through mixed-effects analysis, after which feature dimensionality is reduced according to the cross-subject consistency of fold-wise ReliefF rankings, and Bayesian hyperparameter optimization is compared directly with a fixed baseline using the same subject partitions. Therefore, the main distinction of this study is not the introduction of a new classifier or a claim of superior benchmark accuracy, but an empirically supported procedure for selecting a compact and computationally efficient sEMG processing pipeline for a consumer-grade wearable device in an HRC scenario.

## 3. Methodology

**Figure 1:**
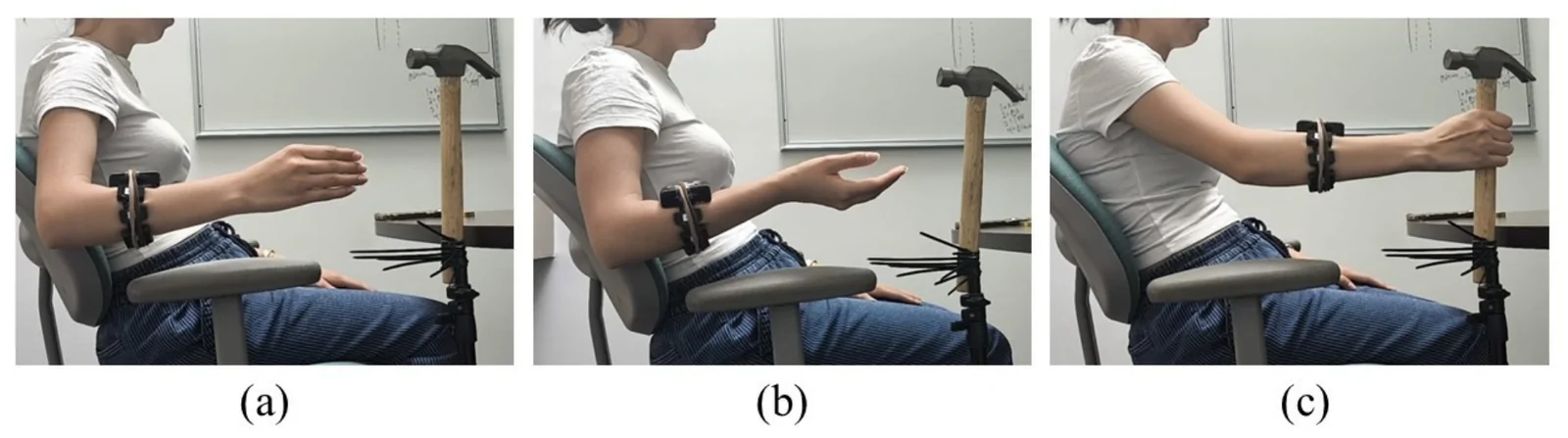
Gestures involved in the research scenario. (**a**) Neutral gesture. (**b**) Ask gesture. (**c**) Take gesture.

Our research scenario is based on an assembly task where an operator and a cobot work interactively toward the same process on the same workpiece. The operator communicates with the robot through gestures to ask for components and tools to assemble the main piece. In this way, three gestures were defined to establish communication between the parties, and they are shown in Figure 1.

1. Neutral: it is the basal gesture. The arm is aligned with the torso, and the forearm forms a 90° angle with respect to the arm.
2. Ask: to ask the robot for a piece or tool. The arm is aligned with the torso, and the forearm forms a 90° angle with respect to the arm in supination.
3. Take: to take the tool reached by the cobot. The arm moves from the initial location in a neutral gesture to take the object placed in a strategic location, and then the fingers open and close to grasp the object.

Five female and five male undergraduate students participated in an activity to gather sEMG and EEG data corresponding to these three gestures. The participants were around 22 years old, right-handed, with similar body constitutions, and without musculoskeletal and neurological disorders. They signed an informed consent and were trained to perform the activity and gestures properly. A research protocol was submitted, revised, and approved by the Tecnologico de Monterrey Institutional Research Ethics Committee; the tracking code for this protocol is P-EIC-202410-003.

**Figure 2:**
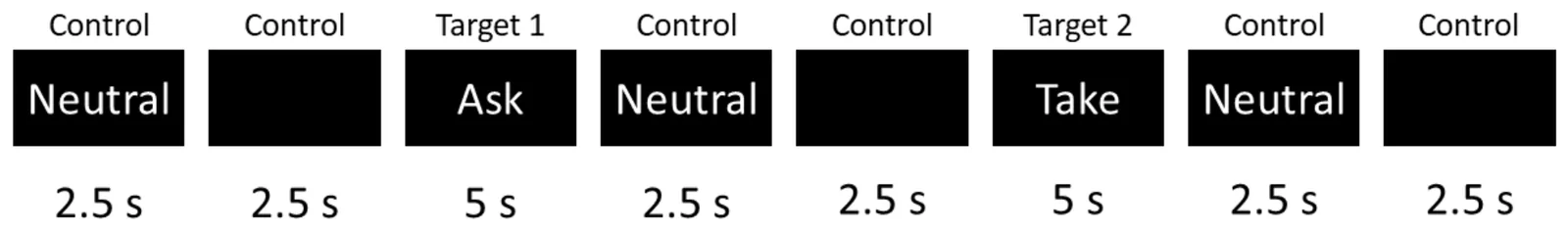
First eight frames of the video for activity 3. Neutral and black windows collect the basal state of the user.

**Figure 3:**
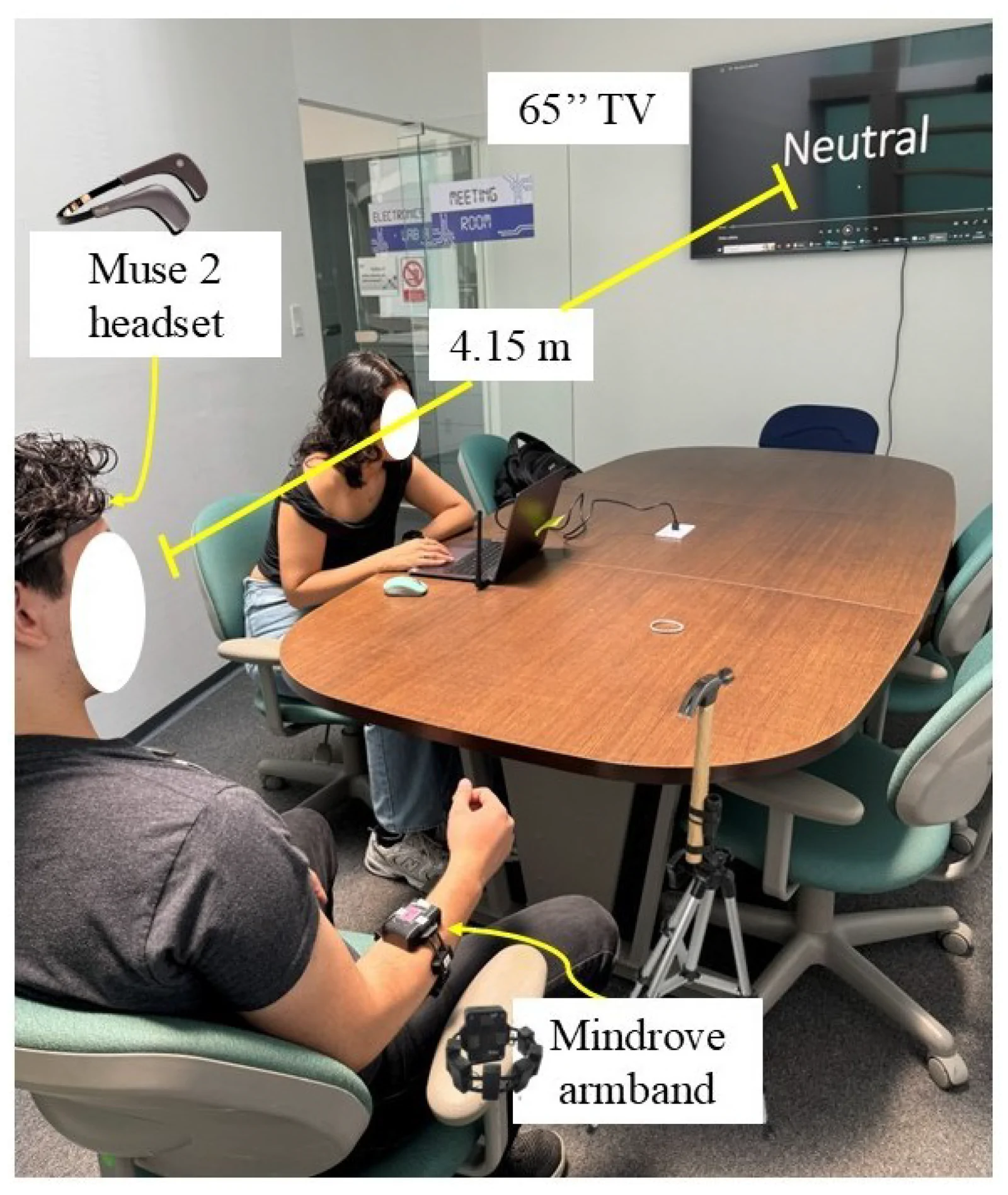
Experimental setup.

The participants performed each of the three gestures according to the instructions given in a video. It showed the words “Neutral”, “Ask”, or “Take”. The words “Ask” and “Take” were randomly shown and kept on screen for 5 s, followed by the “Neutral” word (2.5 s) and a black window (2.5 s), during which the user returned to the neutral gesture to extract their basal state. Figure 2 depicts the first eight video frames. The raw data were labeled according to the gesture indications in the video. The video was reproduced on a 65-inch TV at 4.15 m from the participant; the experimental setup is shown in Figure 3. In addition, a hammer was placed on a tripod to perform the take gesture, as can also be seen in Figure 3.

Participants were asked to concentrate on the activity and to refrain from speaking and moving any part of their body except their forearm. They were allowed to ask questions at any time prior to data collection and abandon the experiment if they wished. The data collection lasted a maximum of 20 min. Personal information, such as demographics and collected biosignals, was treated with absolute confidentiality, and participants were deidentified.

### 3.1. Technological Resources

The wearable device used to collect the sEMG data was the Mindrove armband model ARB.210901 (Mindrove, Budapest, Hungary) with a sampling rate of 500 Hz. It is composed of an inertial measurement unit (IMU) with 6 DOFs and 8 + 2 semi-dry conductive-fabric equidistant electrodes for sEMG; the two additional electrodes are the reference and bias electrodes [48]. Power-line interference was suppressed at acquisition through the built-in notch filter of the Mindrove application, which was enabled prior to data collection; therefore, no additional notch stage was applied during offline processing.

According to a previous study [12], the eight channels were matched to the following forearm muscles: channels 1 through 4 correspond to the flexor carpi radialis, palmaris longus, and flexor carpi ulnaris (channels 3 and 4), respectively, covering the flexor compartment; channels 5 through 8 correspond to the extensor carpi, extensor digitorum, extensor carpi radialis, and brachioradialis, covering the extensor compartment. Due to bodily differences among the participants, channels 3 and 4 could be placed on the flexor carpi ulnaris, or channel 4 could be positioned between the ulna and the flexor carpi ulnaris.

To record EEG data, we used the Muse 2 headset (Muse, Toronto, ON, Canada) with a sampling rate of 256 Hz. It has 4 dry electrodes (TP9, AF7, AF8, TP10), a photoplethysmogram (PPG) sensor, and an IMU with 6 DOFs [49]. The results of the analysis of these data will be reported elsewhere.

Data from sEMG and EEG were recorded using the Lab Streaming Layer (LSL) system (Christian Kothe, San Diego, CA, USA) [50], which centralized the data collection. The activities were also videotaped by the Xiaomi POCO X3 Pro 8GB/256GB smartphone (Xiaomi, Beijing, China), in case of possible concerns during data segmentation.

Data collection and analysis were performed on a Huawei MateBook 14 (Huawei, Shenzhen, China), AMD Ryzen 54600H with Radeon Graphics 3.00 GHz, 16 GB of installed RAM, and a 64-bit operating system. Statistical analyses were carried out in Minitab 22 (Minitab LLC, State College, PA, USA). Data treatments were performed in MATLAB R2025b (MathWorks, Natick, MA, USA), and the classification model was created with the Classification Learner app introduced in MATLAB R2015a.

### 3.2. Data Processing

For the preprocessing of EMG, four binary stages were defined in a fixed execution order: (F) filtering with a fourth-order zero-phase Butterworth low-pass filter at 240 Hz; (O) Hampel outlier removal in windows of 25% of the device sampling rate and three standard deviations; (E) Root Mean Square (RMS) envelope extraction in windows of 100 samples; and (N) Min–Max normalization. The combination of these stages resulted in 16 distinct preprocessing pipelines, as shown in Table 2. Each pipeline was applied to each subject’s own complete time series; in particular, the Min–Max normalization (N) was computed per channel over that subject’s own series, so no scaling statistics were pooled across subjects. Finally, the data were segmented by gesture (Neutral, Ask, and Take), that is, in the 5 s windows corresponding to each of the gestures.

For the filtering, a high-pass stage for low-frequency motion artifacts was deliberately not applied since the forearm rotation in the Ask gesture produces a low-frequency component that marks the start of movement, and removing this band suppressed a waveform feature informative for gesture discrimination. In addition, the zero-phase implementation is non-causal and was applied offline; however, for an actual real-time implementation, a causal Butterworth filter would be necessary, which would introduce the phase distortion that zero-phase filtering is designed to eliminate.

A sliding window approach was employed to extract features of each segmented gesture. Window sizes ranged from 150 to 750 samples (300–1500 ms) in steps of 50 samples, and overlap percentages ranged from 10% to 90% in steps of 10%, producing 117 window-overlap combinations per pipeline. Twelve morphological and statistical features were extracted from each window over the eight sEMG channels, resulting in a total of 96 features. The extracted features include mean, standard deviation (SD), variance (VAR), root mean square (RMS), kurtosis (KURT), modified mean absolute value (MMAV), average amplitude change (AAC), waveform length (WFL), slope sign change (SSC), average energy (AE), skewness (SKEW), and coefficient of variation (CoV). These features were selected based on their effectiveness in capturing discriminative patterns underlying sEMG and EMG signals as widely documented in the literature [51,52,53], but they are also easy to compute and therefore quick to obtain, making them suitable for working with real-time recognition [21]. Features were standardized per subject using z-score normalization to mitigate inter-subject variability.

Per-subject z-score standardization was computed using the unlabeled feature statistics of each participant’s complete recording, including the held-out participant. No class labels from the held-out participant were used for model fitting, feature ranking, or hyperparameter optimization. Nevertheless, this procedure assumes prior access to the target user’s signal distribution and should therefore be interpreted as LOSO cross-subject evaluation with target-specific unsupervised normalization, rather than as a strictly calibration-free protocol.

Table 3 displays their mathematical formulation and provides a brief description of their physical interpretation.

We worked with the Bagged Trees model developed with the Classification Learner of MATLAB with 100 decision trees and a maximum of 20 splits per tree. It is reported that this model has good performance in classifying non-normal [58] and multiclass data [59], it is well-known and powerful in EMG pattern recognition [51].

This study was designed to compare processing-pipeline configurations rather than different classifier families. Therefore, Bagged Trees was maintained as the common classifier throughout all LOSO folds so that changes in performance could be attributed to preprocessing, temporal segmentation, feature selection, and hyperparameter configuration. ReliefF was used exclusively as the feature-ranking algorithm, whereas Bayesian optimization with the Expected Improvement Plus acquisition function was used to tune the Bagged Trees hyperparameters; neither method was treated as an alternative classifier. The direct model-configuration comparison comprised two fixed-parameter baselines (Base All96 and Base Top5) and three Bayesian-optimization configurations (HPO All96, HPO Top5, and HPO Transfer). The algorithms reported in Table 1 provide a contextual comparison with the literature but were not reimplemented because the corresponding studies use different datasets, sensors, gesture sets, and validation protocols.

To ensure the model’s generalization across different users, a Leave-One-Subject-Out (LOSO) cross-validation scheme was adopted. This approach iteratively evaluates the model on an unseen participant in each fold, providing a realistic estimation of the classification performance for new, real-world users.

Since the number of Neutral samples doubles the number of Ask or Take classes, random undersampling was applied to the Neutral class in the training set of each fold to match the size of the minority classes, Ask or Take. A deterministic random seed (rng(test_subject + pipeline_id × 1000) ) was used to ensure reproducibility across folds and pipelines while maintaining independence between experimental conditions.

Within each LOSO fold, feature selection was performed using the ReliefF algorithm (k = 10) [60], which was applied to the balanced training set to rank the 96 features by discriminative relevance. The top 20 features from each fold-specific ranking were then used to train and test the model. The ReliefF ranking was computed exclusively from the training partition of each outer LOSO fold; the held-out participant never contributed to the feature ranking of the fold in which it was tested.

The ReliefF algorithm is a standard statistical filter method that reduces dimensionality by removing redundant or irrelevant features after extraction and is typically used in sEMG and EEG studies due to its low computational cost compared with other methods such as neighborhood component analysis, genetic algorithms, particle swarm optimization, among others [21,61,62].

Considering the recognition accuracy achieved by the classification model trained with the top 20 features, we conducted a mixed-effects analysis of variance (ANOVA) to identify the optimal preprocessing method, window size, and overlap. This pipeline was adopted to perform a dedicated feature selection analysis. This approach was chosen to ensure computational efficiency, thereby avoiding unnecessary processing of less effective combinations.

The ReliefF algorithm was applied within each LOSO fold, preserving the same undersampling seeds to ensure identical data partitions. A consistency analysis was then performed to assess the stability of the selected features across subjects: the consistency score of each feature was defined as the percentage of folds in which it appeared among the top 20 ranked features, reflecting its robustness against inter-subject variability. In case of a tie in consistency score, the feature with the highest mean ReliefF weight across folds was prioritized. Features were subsequently sorted according to this criterion, and the Bagged Trees classifier was trained and evaluated under the same LOSO scheme for each subset size from 1 to 96 features, adding one feature at a time in descending order of consistency and mean weight. This consistency-based elbow analysis determined the optimal number of features by identifying the point at which classification accuracy stabilized without further meaningful improvement. The resulting feature subset was used to train and test the final classification model under the parameters and LOSO scheme already described.

Having established the optimal feature subset, we further investigated whether classification performance could be improved through a systematic hyperparameter optimization (HPO) of the Bagged Trees model using a Bayesian framework. We employed a Bayesian optimizer using the Expected Improvement Plus acquisition function of MATLAB to find the optimal configuration of the Bagged Trees ensemble. The optimization aimed to minimize the classification error through a nested 5-fold cross-validation conducted exclusively on the training partition of each LOSO fold. We defined a search space for three critical hyperparameters using log-scale transformations to ensure a thorough exploration of the lower range values:

- Number of Learning Cycles ($N_{cycles}$): [50,300] (integer, log-scale).
- Maximum Number of Splits ($S_{max}$): [5,200] (integer, log-scale).
- Minimum Leaf Size ($L_{min}$): [1,10] (integer, log-scale).

For each participant fold, the optimizer executed 30 iterations or trials. To ensure reproducibility, all models were initialized with the previously mentioned seed, and the tree learners were set to a reproducible state. Five experimental configurations were compared to disentangle the independent contributions of feature dimensionality and hyperparameter tuning.

1. Base All96: the classifier was trained and tested with fixed hyperparameters ($N_{cycles}=100$, $S_{max}=20$, $L_{min}=1$) using the full 96-feature set.
2. Base Top5: the classifier was trained and tested with fixed hyperparameters ($N_{cycles}=100$, $S_{max}=20$, $L_{min}=1$) using the top 5 features according to their consistency score and weight.
3. HPO All96: Bayesian optimization was applied to the model using the full 96-feature set. Then, it was trained and tested with the same feature set.
4. HPO Top5: Bayesian optimization was applied to the model using the top-5-feature set. Then, it was trained and tested with the same feature set.
5. HPO Transfer: The optimized model resulting from the HPO All96 procedure was trained and tested with the top-5-feature set.

**Figure 4:**
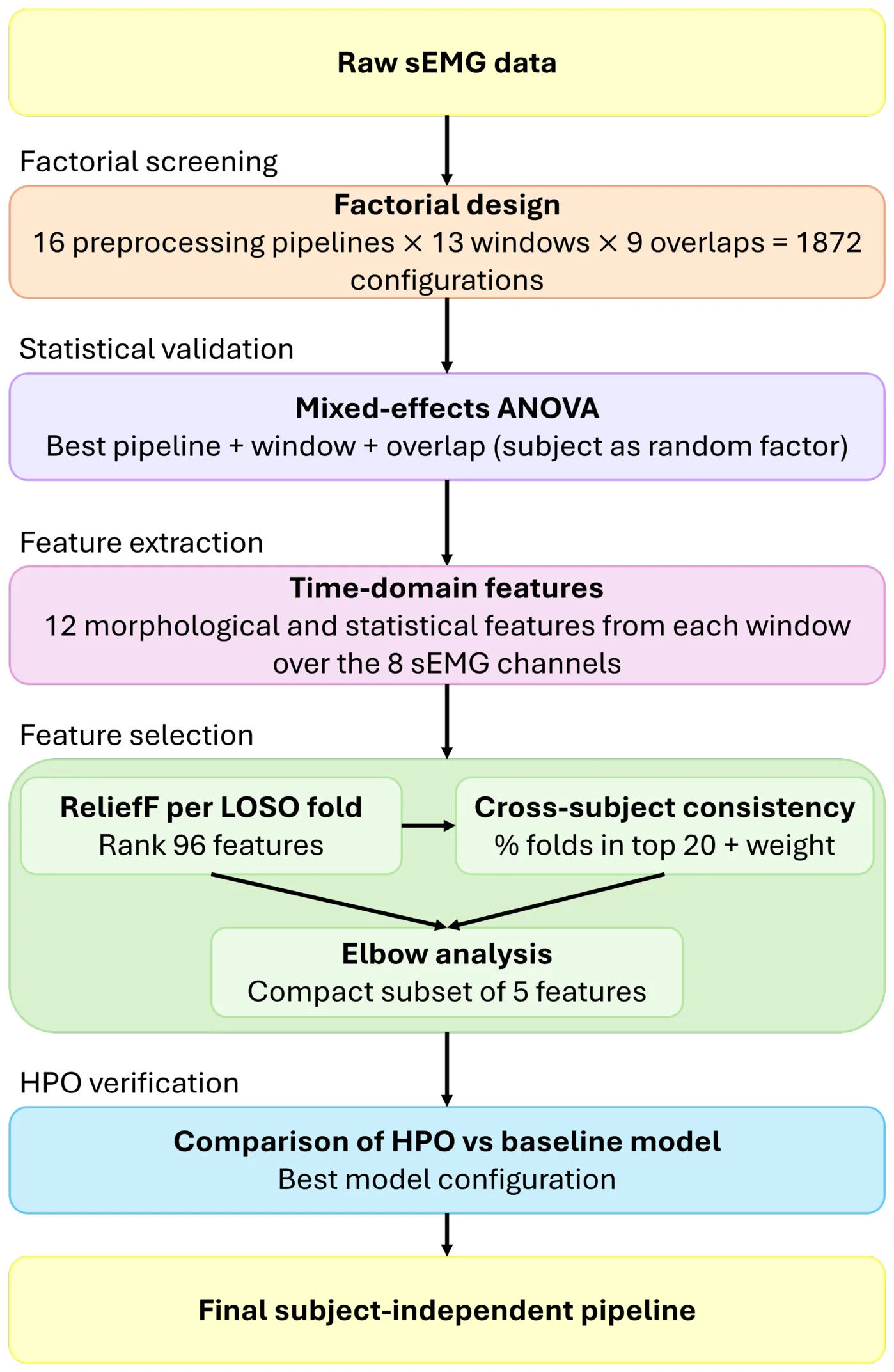
Overview of the methodological workflow. Raw sEMG data undergo a factorial screening stage evaluating 1872 configurations (16 preprocessing pipelines × 13 window sizes × 9 overlap levels) under LOSO cross-validation. A mixed-effects ANOVA statistically validates the best-performing pipeline, window size, and overlap, treating subject as a random factor. Twelve time-domain features are then extracted per window across the 8 sEMG channels (96 features total). Feature selection combines ReliefF ranking within each LOSO fold with a cross-subject consistency score, followed by an elbow analysis that identifies a compact 5-feature subset. Finally, Bayesian hyperparameter optimization (HPO) is compared against the fixed-hyperparameter baseline to verify whether tuning yields additional gains, producing the final subject-independent pipeline.

Figure 4 summarizes the overall data processing workflow described in this section, from the factorial experimental design to the final subject-independent pipeline obtained after feature selection and hyperparameter optimization verification.

### 3.3. Statistical Analysis of Classification Performance

In order to determine the processing pipeline that maximizes the recognition accuracy of the three gestures, a mixed-effects analysis of variance (ANOVA) was implemented with a significance level of α=0.05. We treated as fixed factors the preprocessing methods and the sliding window parameters (size and overlap percentage) used for feature extraction, while the subject was included as a random factor to account for inter-subject variability.

Effect sizes were reported using generalized eta-squared ($\eta_{G}^{2}$), described in (1), as it provides a less biased measure in complex and mixed-design ANOVA models. This metric is particularly appropriate when both fixed and random factors are present, as it accounts for variance associated with random effects such as inter-subject variability [63]. Post hoc comparisons were performed on pipeline, window size, and overlap using Tukey’s test with a significance level of α=0.05.

(1) $$\eta_{G}^{2}\;=\;\frac{SS_{\mathrm{effect}}}{SS_{\mathrm{effect}}+SS_{\mathrm{error}}+SS_{\mathrm{subject}}}$$

The ANOVA assumptions were evaluated through residual diagnostics, including normal probability plots, histograms, and residuals versus fitted values. The residuals showed an approximately normal distribution, with minor deviations in the tails. Homogeneity of variance was generally satisfied, as the residuals versus fitted values plot did not show a clear cone-shaped pattern. No clear patterns were found in the residuals over the observation order, suggesting independence at the global level.

Given the exhaustive combinatorial nature of the experimental design, with 1872 configurations tested on the same set of recordings, the induced dependence structure reduces the effective degrees of freedom relative to the nominal ones, which in turn inflates the apparent statistical significance (*p*-values). Consequently, to mitigate the risk of overestimating significance due to these repeated measures, our interpretation of the mixed-effects ANOVA is based on the generalized eta-squared effect sizes. This approach ensures that the identified differences in the preprocessing pipelines represent meaningful impacts on system accuracy rather than marginal statistical artifacts.

In addition to the primary factorial analysis, two secondary mixed-effects comparisons were conducted. First, three five-feature representations (the subset selected directly by the consistency-based ReliefF ranking, an AAC-based substitute, and a WFL-based substitute) were compared under the same LOSO protocol. Feature-set configuration was treated as a fixed effect and subject as a random effect; each set was evaluated in three repetitions using different undersampling seeds. Second, the five feature–hyperparameter configurations (Base All96, Base Top5, HPO All96, HPO Top5, and HPO Transfer) were compared using configuration as a fixed effect and subject as a random effect. For both comparisons, the significance level was set to α=0.05, and the assumptions of normality, homogeneity of variance, and independence were evaluated using normal probability, residual-versus-fitted, and residual-versus-order plots. These analyses were designed to determine, respectively, whether AAC could be replaced by the computationally simpler WFL representation without a statistically detectable loss of accuracy and whether Bayesian hyperparameter optimization provided a statistically detectable improvement over the fixed baseline.

## 4. Results

**Figure 5:**
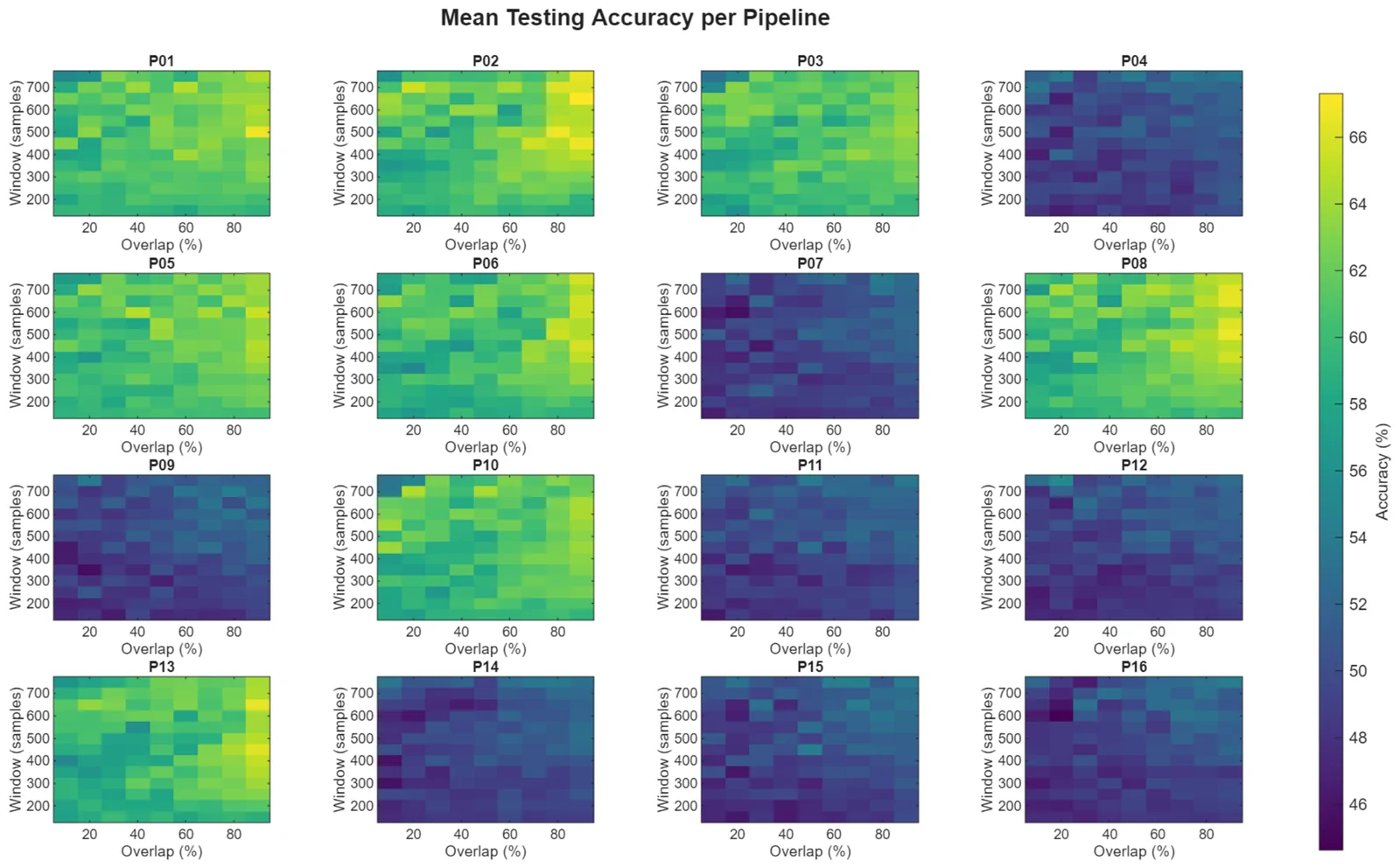
Mean LOSO-CV testing accuracy (%) as a function of window size (samples) and overlap (%) for each of the 16 sEMG preprocessing pipelines (P01–P16).

The testing accuracy means used to perform mixed-effects ANOVA and determine the optimal configuration of pipeline, window size, and overlap percentage are shown in Figure 5, where a clear stratification of performance is observed among the evaluated pipelines. Pipelines incorporating envelope computing (4, 7, 9, 11, 12, 14, 15, and 16) consistently achieved lower mean accuracy (<50%) across a wide range of segmentation parameters, while the rest of the pipelines seem to achieve a similar accuracy; this effect is attributed to the reduction in signal dynamics due to the softening of the envelope.

On the other hand, the effect of window size and overlap percentage is not as pronounced as that of the preprocessing pipeline; however, there is a trend: as both parameters increase, the color gradient of heatmaps shifts to warmer hues, which corresponds to higher accuracy values. This behavior suggests that wider windows capture greater temporal context of the sEMG signal, improving discriminability between gestures, while high overlap increases training sample density, although the resulting windows are partially correlated.

A mixed-effects ANOVA was conducted to quantitatively assess the relative contribution of each factor, and the results are presented in Table 4. It is confirmed that the preprocessing pipeline is the most influential factor affecting classification performance ($\eta_{G}^{2}=0.320$, p<0.001), followed by inter-subject variability ($\eta_{G}^{2}=0.296$, p<0.001). In contrast, window size ($\eta_{G}^{2}=0.010$) and overlap ($\eta_{G}^{2}=0.016$) exhibit statistically significant but smaller effects.

While the interactions do not produce a statistically significant change, with the exception of the interaction between window size and overlap ($\eta_{G}^{2}=0.005$, p=0.002), the size of their effect is negligible. These results have a direct practical implication: given the absence of significant interactions, the optimization of preprocessing pipelines and segmentation parameters can be treated as independent problems, thus reducing the search space in future experimental designs.

**Figure 6:**
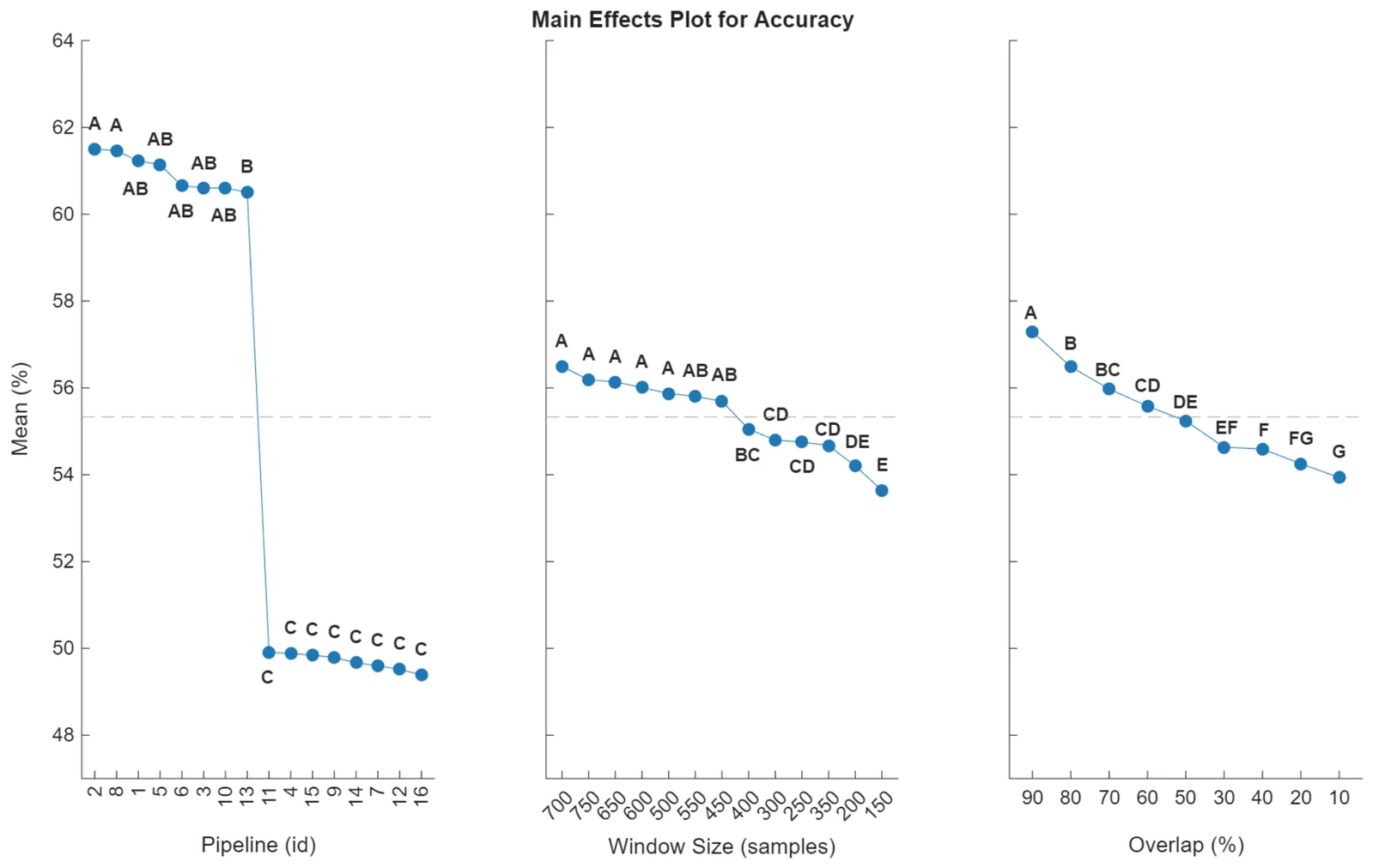
Main effects plot for testing classification accuracy (%) across three experimental factors: preprocessing pipeline configuration (id), segmentation window size (in samples), and window overlap percentage. Each point represents the marginal mean accuracy averaged over all combinations of the remaining factors. The dashed horizontal line indicates the grand mean (55.27%). Means sharing at least one letter are not significantly different (Tukey HSD, α=0.05).

The main effects plot with Tukey grouping (Figure 6) illustrates the marginal mean accuracy for each factor level. Regarding the preprocessing pipeline, two statistically distinct clusters are evident: a high-performance group comprising pipelines 2, 8, 1, 5, 6, 3, 10, and 13 (Tukey groups A–B, mean above 60%), and a low-performance group formed by the remaining pipelines (Tukey group C, mean below 50.0%), which was discussed previously.

With respect to window size, Tukey groupings reveal that windows of 450 samples and above belong to the same statistical group (A), indicating that accuracy does not differ significantly within this range, i.e., selecting a window of 750 samples yields no statistically meaningful advantage over 450 samples. Nevertheless, below this threshold, the performance of the classifier begins to decrease significantly. This suggests that 450 samples represents a practical lower bound for reliable gesture discrimination with the proposed feature set, while further increases in window size offer no additional benefit.

For the overlap factor, no practical lower bound analogous to that observed for window size can be established. Instead, accuracy increases with overlap percentage, and each level forms a distinct Tukey group (A through G), indicating that any reduction in overlap carries a statistically detectable cost in classification performance. Nonetheless, the practical magnitude of this effect remains modest, spanning 3.3 percentage points across the full range of overlap levels tested (10–90%), with a difference of only 1.71 percentage points between the top-performing group A (90% overlap) and group C (60% overlap). Therefore, in scenarios where computational resources are limited, an overlap of 60% may represent a computationally efficient alternative.

Based on these findings, the best 10-performing combinations of preprocessing and segmentation parameters were identified in Table 5. The highest accuracy (67.31%) was achieved using pipeline 2 (filtering) with a window size of 650 samples and 90% overlap. The configurations share common characteristics: filtering as a core preprocessing step, high overlap (≥80%) and medium-to-large window sizes (450–750 samples), which is consistent with the results of the Tukey test. In contrast, more complex preprocessing chains involving multiple stages did not yield additional improvements. It could be concluded that, within the evaluated configuration space, the best performance is obtained with simple preprocessing strategies combined with appropriate segmentation parameters. For practical purposes, the best configuration (rank 1) was retained for the rest of the analysis.

In addition, accuracy and macro F1-score are in close agreement, with differences ranging from 0.05 to 0.83 percentage points. This suggests that the model achieved balanced performance across the three gesture classes and that the random undersampling approach mitigated the class imbalance introduced by the higher proportion of Neutral samples, preventing the model from being biased toward the majority class.

**Figure 7:**
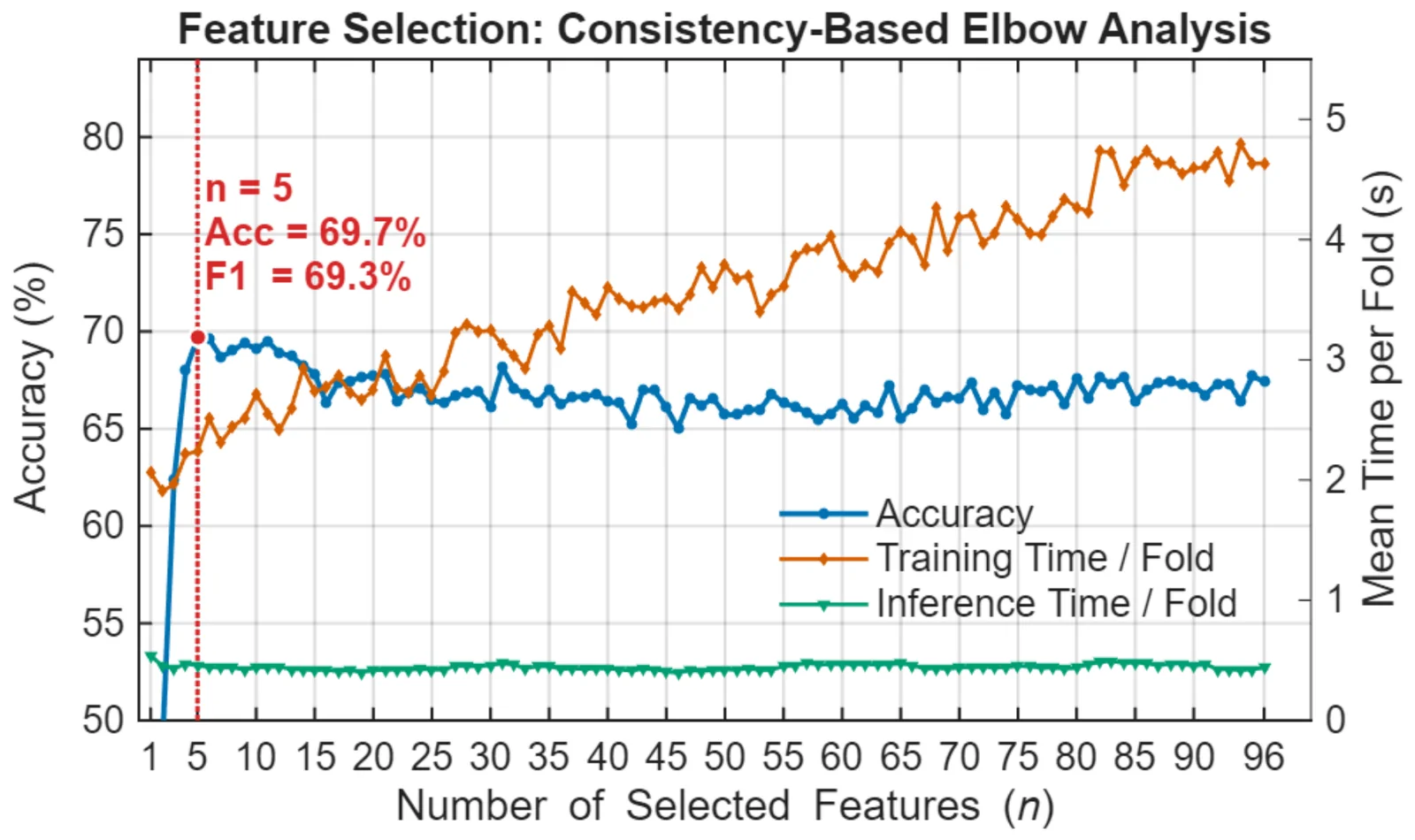
Elbow analysis for consistency-based feature selection under LOSO CV using a Bagged Trees classifier. The left axis shows mean testing accuracy (%) over 10 subjects as a function of the number of selected features (*n*), where features are added in descending order of consistency score and mean ReliefF weight. The right axis shows mean training time and inference time per fold (s), measured as CPU time. The selected operating point (n=5) is indicated by a dashed vertical line.

Following the feature selection analysis to determine the minimum number of features required to maximize classification performance, Figure 7 reveals an elbow in the accuracy curve at n=5 features, where the classifier achieves its peak performance of 69.7% testing accuracy and 69.32% macro F1-score, surpassing the full 96-feature baseline in accuracy (67.41%) while remaining similar in F1-score (67.67%). Beyond this point, incorporating additional features yields no performance gain.

From a computational standpoint, training time increased with dimensionality, from 2.24 s at n=5 to 4.63 s at n=96, representing a 51.62% reduction in training cost per fold. In contrast, inference time remains practically flat across all feature subset sizes, which is consistent with the tree-traversal mechanism of Bagged Trees ensembles: prediction cost is primarily determined by tree depth and ensemble size, not by input dimensionality.

Consequently, the selection of n=5 features reduces the feature space by 94.79% while simultaneously improving classification accuracy and reducing training overhead, without any penalty on inference latency.

We acknowledge that the number of features was determined by evaluating all subset sizes on the aggregated cross-validation results, which may introduce a mild optimistic bias. To verify robustness, we applied a rule of selecting the smallest number of features whose accuracy lies within 1% of the maximum accuracy of the curve, which corresponds to 5 features. However, a fully nested selection within each training fold remains a refinement for future work.

**Figure 8:**
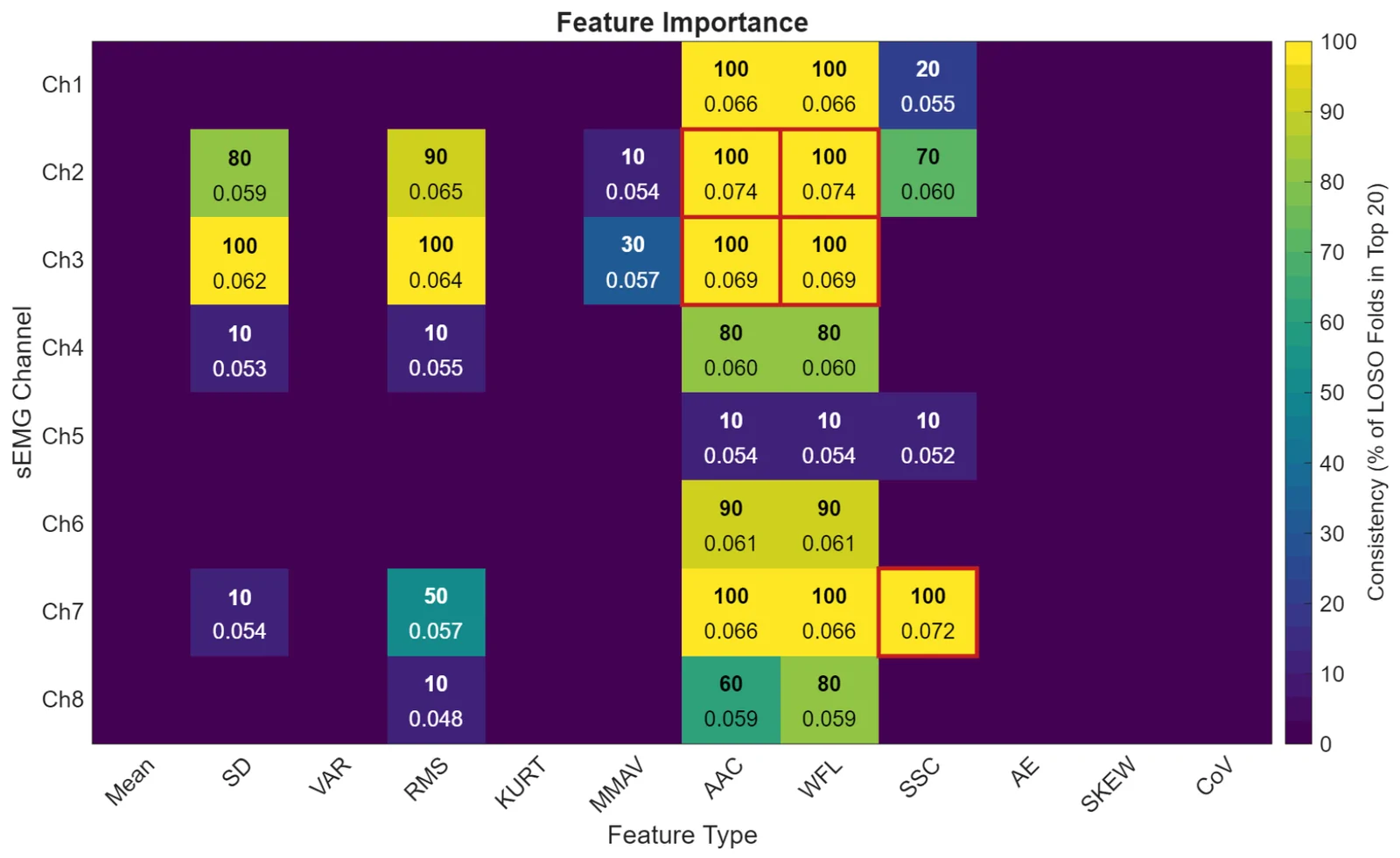
Consistency heatmap of ReliefF-based feature selection across LOSO CV folds. Each cell displays the consistency score (% of folds in which the feature appeared in the top 20, upper value), and the mean ReliefF weight across folds (lower value). Empty cells indicate features that never appeared in the top 20 across any fold. Red-bordered cells highlight the top 5 most consistent features selected with the higher ReliefF weight as the optimal subset (accuracy = 69.7%). Color intensity reflects consistency, from low (dark purple) to high (yellow).

The 5 features with which a 69.7% accuracy was achieved are shown enclosed in red in Figure 8, corresponding to AAC and WFL from channels 2 and 3, and SSC from channel 7. Note that this is the subset selected directly by the ranking algorithm proposed here. The AAC and WFL features exhibit the highest consistency, appearing in the top 20 in 100% of LOSO folds across channels 1, 2, 3, and 7, with mean ReliefF weights ranging from 0.066 to 0.074. The SSC feature from channel 7 also achieves 100% consistency, and its weight is higher than the others (0.072), while SD and RMS show moderate consistency concentrated in channels 2 and 3. In contrast, features such as Mean, VAR, KURT, AE, SKEW, and CoV contribute negligibly across all channels, suggesting low discriminative power for the recognition of the three gestures.

Given the mathematical similarity between AAC and WFL, two additional feature sets were evaluated to determine whether one could be substituted for the other without loss of classification performance. The first set comprised the four AAC features with 100% consistency from channels 1, 2, 3, and 7 and SSC from channel 7; the second replaced AAC with the corresponding WFL features under the same channel selection and SSC from channel 7.

The three sets were evaluated under the same LOSO scheme with three repetitions per set, using a mixed-effects model with subject as random factor. The assumptions of normality, homogeneity of variance, and independence were fulfilled according to residual plot analysis. For each repetition, the seed was modified per repetition to introduce variability in the random undersampling of the Neutral class. The analysis revealed no statistically significant difference in accuracy between the sets (F(2,78)=0.34, p=0.712, α=0.05), confirming that AAC and WFL are interchangeable in this classification context. Along with SSC from channel 7, WFL from channels 1, 2, 3, and 7 were selected as the feature set for the Base Top5 model (accuracy = 69.86% with the original seed) as its cumulative formulation requires fewer arithmetic operations per window, which is desirable for embedded real-time implementation where computational resources are limited. Since AAC and WFL are mathematically almost identical, their similar performance is expected.

After finding the optimal subset of features (n=5), it was investigated whether performance could be improved by Bayesian hyperparameter optimization (HPO). The results of the five configurations evaluated under the LOSO scheme are presented in Table 6. Given the marginal change in accuracy, to determine with certainty which configuration produced better accuracies, an ANOVA of mixed effects was performed, with the subject as random factor and the configuration as fixed effect. The assumptions of normality, variance, and independence were satisfied according to the diagnosis of residuals.

The ANOVA demonstrated that the choice of configuration did not exert a statistically significant effect on accuracy (F(4,36)=1.27, p=0.298, α=0.05). While the HPO All96 configuration attained the highest mean accuracy (70.55%), above the Base Top5 configuration (69.86%), this difference did not reach statistical significance. Given that there were only ten participants, the analysis may lack sufficient power to detect a modest improvement. Therefore, this result should be read as the absence of a detected difference rather than as evidence that hyperparameter optimization offers no benefit.

From a practical implementation perspective, and despite the marginally higher mean accuracy of HPO All96, we retain the Base Top5 configuration on the grounds of computational efficiency and parsimony rather than maximizing the accuracy: it achieves a comparable accuracy of nearly 70% while using only five features, and requires no hyperparameter optimization cycles. In addition, it reduces inter-subject variability from 12.99 with the Base All96 to 12.53, indicating that eliminating redundant features reduces noise and improves model performance.

Table 7 presents the generalization capability of the optimal configuration (Base Top5) per participant. The model achieved a mean accuracy of 69.86% and 69.59% of F1-score, confirming that it maintains consistent performance across gesture classes. The close agreement between the mean accuracy and F1-score suggests broadly balanced aggregate performance across classes, although the class-level analysis revealed greater confusion between Ask and Neutral.

However, there is a high degree of inter-subject variability; in fact, performance ranged from a maximum accuracy of 85% (subject 9) to a minimum of 52.59% (subject 6). While the three lowest accuracies (<60%) occurred in female participants (subjects 4, 6, and 8), the highest accuracy in the entire study was also achieved by a female participant (subject 9). Although it is a noteworthy pattern, with such a small sample, it is not possible to reliably determine whether the model’s performance depends on the sex of the participant.

Consistent with the literature, the variability in model performance is due to physiological differences; for example, increased subcutaneous fat thickness attenuates the signal. Whereas a high Body Mass Index (BMI) is typically associated with a thicker adipose layer, our results do not demonstrate a direct correlation between BMI and classification accuracy; in other words, the available data do not allow for a definitive assessment of the specific impact of adipose tissue across participants. Nevertheless, each electrode was intended to be placed in a specific muscle, but the fixed geometry of the armband restricts individualized electrode alignment; therefore, there are slight variations in electrode placement that may influence the model’s performance.

The mean specificity (83.81%) indicates a relatively low false-positive rate, which is desirable for minimizing unintended activations. The mean sensitivity (70.18%) indicates that approximately 70% of class-specific windows were correctly detected; most remaining errors involved confusion between Ask and Neutral.

In order to consolidate the accuracy values reported throughout this section and summarize the information, Table 8 traces each figure to its configuration, feature set, and 95% confidence intervals over the ten LOSO folds. The three full-feature values correspond to distinct models: 67.31% is the best combination of the factorial search evaluated with the fold-wise top-20 features, whereas 67.41% and 68.10% both use the full 96-feature set under identical LOSO partitions and the same undersampling seed, and their difference arises from the tree-learner specification; the Elbow All96 uses the default leaf size of Matlab, while Base All96 specifies the min leaf size as 1, this produces different base trees.

Similarly, the two five-feature configurations reflect that 69.70% corresponds to the subset that maximizes mean LOSO accuracy in the consistency-based ranking (which includes AAC terms), and 69.86% to the final model obtained after substituting AAC with the equivalent WFL features, an interchange justified by the non-significant AAC and WFL comparison. As shown, the confidence intervals overlap, which aligns with the absence of statistically significant differences among the configurations described above, and highlights that the estimates should be interpreted with caution given the limited sample size of ten participants.

**Figure 9:**
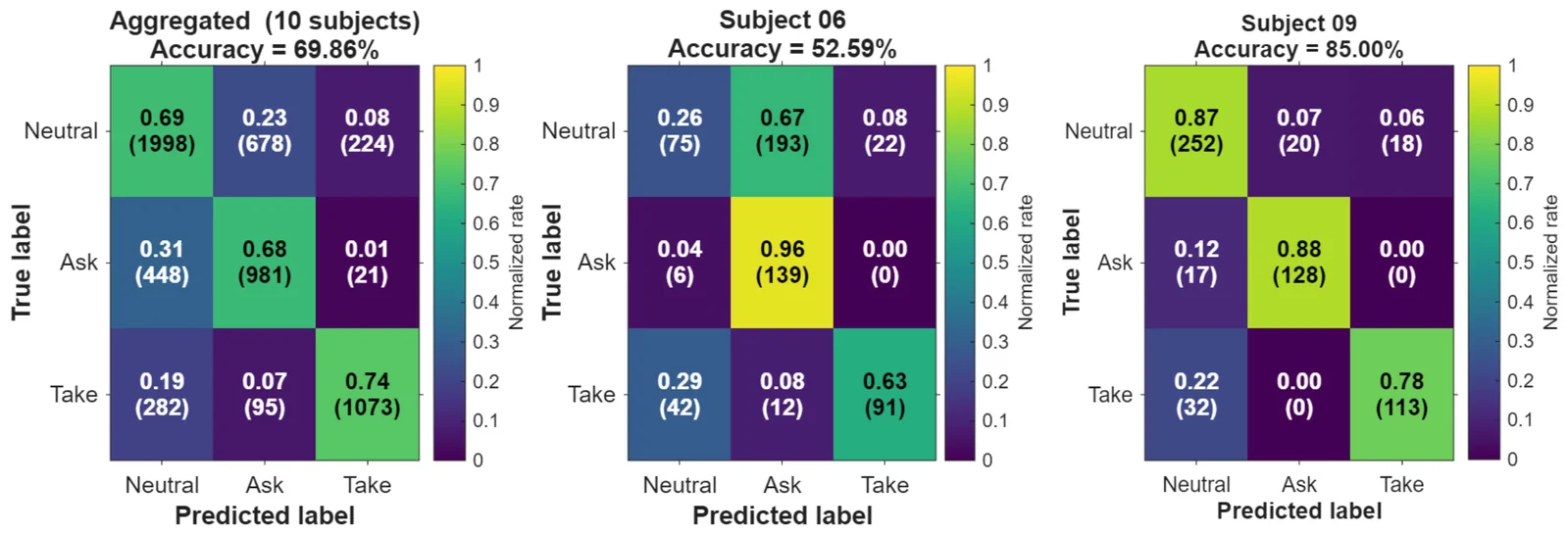
Normalized confusion matrices for the Base Top5 configuration across three gesture classes (Neutral, Ask, Take). (**Left**) Aggregated matrix summed over all 10 subjects (overall accuracy = 69.86%); cell annotations show the normalized rate and the absolute window count in parentheses. (**Center**) Per-subject matrix for Subject 06 (accuracy = 52.59%), the lowest-performing participant, exhibiting strong bias toward the Ask class (recall = 0.96) and poor Neutral discrimination (recall = 0.26). (**Right**) Per-subject matrix for Subject 09 (accuracy = 85.00%), the highest-performing participant, with consistently high recall across all three classes.

Analyzing in detail the confusion matrices presented in Figure 9, the gestures that are often confused are Ask and Neutral. In the aggregate matrix, it is shown that 23% of Neutral gestures are classified as Ask and 31% of Ask gestures are classified as Neutral, which indicates Ask and Neutral share similar patterns and the electromyographic signature of Ask is less intense or more similar to resting muscle tone. In contrast, Take has a better-defined pattern, which is confirmed by the 74% success rate.

Regarding the confusion matrix of subject 6 (the worst case), Ask has an almost perfect recall of 96%, but 67% of the time the subject is in Neutral and the model classifies it as Ask; therefore, it can be said that the model is biased to Ask gesture. On the other hand, subject 9 presents the best metrics, which shows that it is possible to recognize the three classes with the computed features. While the preprocessing pipeline proved effective for subject 9, subject 6 exhibited higher noise levels that were not fully mitigated during the preprocessing stage; this also explains the differences in the performance metrics.

Table 9 reports the per-class metrics for the Base Top5 configuration, which confirms that Ask is the least separable class (precision = 55.93%) and it is frequently confused with Neutral, whereas Take is the best recognized gesture (F1 = 77.53%), consistent with its more distinct activation pattern. These metrics are computed at the window level; given the 90% overlap, adjacent windows are not independent, so the reported counts correspond to classifications of overlapping windows rather than distinct, independent gesture attempts.

## 5. Discussion

This study aimed to address three research questions focused on the systematic optimization of sEMG processing pipelines for subject-independent gesture recognition, where the influence of preprocessing, temporal segmentation, feature selection, and hyperparameter optimization was evaluated. The results offer evidence-based insights that call into question several widespread assumptions in the field.

The mixed-effects ANOVA confirmed that the selection of the preprocessing pipeline represents the most influential factor in classification accuracy (p<0.001, $\eta_{G}^{2}=0.32$) under the conditions specified in this work. This is important because previous work has largely agreed on a nearly standard sequence of operations: filtering, rectification, envelope extraction, and normalization [14,15,18].

A critical finding is the performance degradation (accuracy < 50%) observed in all pipelines that incorporated RMS envelope extraction (pipelines 4, 7, 9, 11, 12, 14, 15, and 16), which we attribute to the smoothing operation that suppresses the transient waveform dynamics that carry discriminative information, although envelopes remain standard for estimating muscle activation intensity or movement onset in sEMG [35,36].

The optimal preprocessing configuration consisted solely of basic low-pass filtering, while more intricate processing sequences do not provide any further advantage, an outcome consistent with the wearable and lightweight processing philosophy favored for real-time human–machine interaction [27]. In addition, our results are consistent with the growing body of recent research on gesture recognition that processes raw [37,38,42,64] or merely filtered signals [25,27,65] rather than extracting an envelope, since maintaining the nonlinear complexity of the signal [8] contributes more to classification performance, which was our objective, than tracking the activation profile.

Regarding temporal segmentation, the window size and overlap reached statistical significance (p<0.001), but their effect sizes were an order of magnitude smaller ($\eta_{G}^{2}=0.01$ and $\eta_{G}^{2}=0.016$ respectively) than the other factors. This contrasts with the attention these parameters receive in the segmentation literature [19,66]. However, the lack of significant interaction terms indicates that preprocessing and segmentation may be optimized separately, reducing the search space for future work.

The absence of statistically significant differences among window sizes from 450 to 750 samples (900–1500 ms at 500 Hz) indicates that there is sufficient temporal context for a reliable classification from 450 samples. Nevertheless, our operating point is above the 200 to 300 ms range often reported as optimal for latency-critical applications such as prostheses [40]. This discrepancy with the state of the art may be due to the low resolution of our consumer-grade device, which restricts the observable spectrum to a Nyquist frequency of 250 Hz, and provides fewer samples per window than the high-density and high-resolution devices used in other studies, where sampling rates of 1 to 2 kHz are common [29,41,67,68].

The continuous performance increase observed with higher overlap percentages (reaching maximum accuracy at 90%) highlights the need for closely spaced training boundaries to capture transitions between gestures, even at the cost of greater data redundancy. The lower bound of the overlap percentage set at 60% provides a near-optimal trade-off and is in agreement with the overlap values of 50% to 75% usually adopted in the literature [25,41,69,70,71]. It is important to note that, with 90% overlap, the window of 650 samples produces a new prediction every 130 ms, which supports responsive interaction even though each prediction integrates 1.3 s of signal. A full end-to-end analysis of latency is deferred to future work in real-time settings.

Regarding feature selection, our methodology allows us to identify persistent features across LOSO folds, but also to calculate their ranking magnitude based on the ReliefF algorithm, which is widely applied in research due to its low computational cost [21,61,62]. This dual filter aims to enhance robustness to inter-subject variability. The resulting five-feature subset reduced dimensionality by 94.79% while slightly improving accuracy over the full 96-feature set, confirming that an extensive set of features can introduce redundancy that may degrade rather than aid performance [19,23,25].

The most discriminative features were the average amplitude change (AAC), waveform length (WFL), and slope sign change (SSC), and these align with previous studies that have identified them as dominant over other time-domain features [25,51,55]. The lack of a statistically significant difference between AAC and WFL (p=0.712) enabled us to choose WFL based on its small number of arithmetic operations per window compared with AAC, which is a benefit for embedded applications [43,64].

The results obtained under LOSO training suggest that inter-subject variability is a leading factor limiting system performance, since the recognition accuracy ranges from 52.59% (subject 6) to 85% (subject 9). This particularity causes hyperparameter optimization to yield results that do not significantly improve the model under the Base Top5 configuration. Although the literature reports that tree-based ensembles capture nonlinear relationships in sEMG [22,23,51] and achieve accuracies of up to 98% in some settings [72], this divergence is attributed to instrumental factors such as residual noise inadequately removed by preprocessing in certain subjects, and the fixed armband geometry does not allow customized alignment of the electrodes with individual muscles.

For benchmarking context, the final Base Top5 configuration achieved a mean LOSO accuracy of 69.86%. Lin et al. reported accuracies of 87.03% and 94.53% under a calibration-assisted LOSO protocol that used one normalization cycle from the target user [45]. Under a stricter calibration-free protocol, Yang et al. obtained 89.4% on NinaPro DB2 and 86.9% on CapgMyo DBa [46]. Higher values have also been reported under subject-specific or predefined benchmark partitions: Peng et al. achieved 95.23% mean intra-subject accuracy and 95.72% in online interaction [44], whereas Zhang et al. reported accuracies ranging from 81.47% to 98.95% across three benchmark datasets [41]. These results should not be interpreted as a direct ranking because the studies differ in gesture vocabulary, number of participants, sensor density, sampling rate, preprocessing, and validation protocol. In contrast to approaches primarily intended to maximize benchmark accuracy, the contribution of the present study is the controlled evaluation of 1872 processing configurations using an eight-channel consumer wearable, together with a 94.79% reduction in feature dimensionality and a 51.62% reduction in training time.

### Limitations and Future Work

Several limitations should be considered when interpreting these results. First, this study is based on a small and homogeneous sample of ten participants recorded in a single session; this constrains the generalizability of the findings and prevents a conclusive evaluation of factors such as the impact of participant sex or adipose tissue on classification accuracy. In future work, we intend to address robustness to electrode repositioning or to signal variation across repeated sessions and broader populations, along with subject-adaptation methods.

Second, although class labels from the held-out participant were excluded from model fitting, feature ranking, and hyperparameter optimization, the Min–Max and z-score transformations used unlabeled statistics from that participant’s complete recording. Therefore, the protocol should be interpreted as LOSO with target-specific unsupervised normalization rather than as strictly calibration-free evaluation. Furthermore, although the ReliefF ranking was calculated exclusively from the training partition of each outer fold, the final number of features was selected from the accuracy curve aggregated across all LOSO folds, which may introduce optimistic bias. Future work will employ normalization parameters derived exclusively from the training participants and a fully nested procedure for selecting the feature-subset size.

A limitation of the current statistical evaluation lies in the strict dependence structure of the data, as multiple pipeline configurations (1872) were iteratively tested on identical raw recordings. While these repeated measures may inflate the F-statistics of the mixed-effects ANOVA, the magnitude of the observed effects validates our conclusions, with the effect size of the preprocessing pipeline vastly outweighing the impact of window size or overlap.

Furthermore, the substantial decrease in classification accuracy when RMS envelope extraction was applied suggests that this effect is practically meaningful rather than a marginal statistical artifact. Nevertheless, the dependence-induced inflation of *p*-values remains a limitation; therefore, our interpretation emphasizes effect sizes rather than nominal significance. Future work will use a repeated-measures model with subject-by-condition terms or a participant-level permutation test to provide a more robust assessment of statistical significance.

We also note that the preprocessing pipeline was modeled as a single 16-level factor rather than as four separate binary factors (F, O, E, N); an explicit binary-factor model would more directly quantify the individual and interactive contribution of each operation. Developing a binary-factor formulation of the preprocessing operations is recognized as an aspect that will be further refined in subsequent work.

Because Bagged Trees was maintained as the sole classifier family, the observed effects of the processing pipeline remain conditional on this model; replication of the factorial evaluation using classifiers such as LDA, SVMs, k-NNs, and neural architectures constitutes an important direction for future work.

Finally, a full end-to-end latency analysis under real-time operating conditions needs to be performed. Future work will also focus on mitigating inter-subject variability through subject-adaptation and transfer-learning methods, enabling the model to be customized for each new user with minimal calibration data. The fusion of sEMG with complementary biosignals such as EEG will be explored to improve the distinction between gestures that share similar muscle-activation patterns, with a particular focus on the commonly confused Ask and Neutral categories.

## 6. Conclusions

The purpose of this research was to assess different processing pipelines for the recognition of three hand gestures (Neutral, Ask, Take) using a Bagged Trees model trained and tested with sEMG data under a LOSO scheme across ten participants. In a first iteration, three factors that can influence recognition accuracy were tested: the preprocessing pipeline, window size, and overlap percentage. Through a mixed-effects ANOVA, all three were found to be significant, but the preprocessing pipeline had a greater influence ($\eta_{G}^{2}=0.320$) on accuracy compared with window size ($\eta_{G}^{2}=0.010$) and overlap percentage ($\eta_{G}^{2}=0.016$). Remarkably, every pipeline that included RMS envelope extraction reduced accuracy below 50%, so the best setup used only basic low-pass filtering, demonstrating that simple preprocessing can outperform more complex ones.

Once the optimal combination of preprocessing pipeline and window-segmentation parameters was identified, ReliefF-based consistency analysis reduced dimensionality by 94.79% while improving accuracy from 68.10% to 69.86% and reducing training time by more than 50%. The final compact subset comprised WFL from channels 1, 2, 3, and 7 and SSC from channel 7. Bayesian hyperparameter optimization yielded no statistically significant gain over this baseline, together with the wide accuracy range across subjects (52.59–85.00%), suggests that differences between subjects are a primary factor constraining performance, a hypothesis that warrants confirmation in larger cohorts.

The close agreement between mean accuracy and F1-score indicates broadly balanced aggregate performance, although Ask and Neutral remained the most frequently confused classes. The mean specificity of 83.81% indicates a relatively low false-positive rate at the window level, while the mean sensitivity of 70.18% indicates that approximately 70% of class-specific windows were correctly detected. Beyond these results, the study contributes a reproducible data-collection protocol for three HRC-relevant gestures, with interleaved neutral and rest periods. As detailed in Section Limitations and Future Work, future work will address inter-subject variability and explore the fusion of sEMG with complementary biosignals such as EEG.

## Figures and Tables

**Table 1:** Quantitative comparison of representative sEMG gesture recognition studies. Reported values correspond to the original evaluation protocols and should not be interpreted as directly comparable rankings.

Study	Data and Evaluation	Method	Reported Quantitative Result	Comparability Implication
Jie et al. [25]	Eight subjects, 16 channels, subject-specific repeated trials	Two-stage PSO and weighted k-NN variants	Weighted variants improved mean accuracy by approximately 4 percentage points over PSO–k-NN; channel optimization added 0.70 and 0.13 points for PSO–WRKNN and PSO–WLMRKNN, respectively.	Within-subject evaluation; it does not demonstrate performance on unseen subjects.
Karnam et al. [43]	Five benchmark datasets	Hybrid CNN–BiLSTM (EMGHandNet)	Accuracies of 95.77%, 95.90%, 91.65%, 91.29%, and 98.33% on NinaPro DB1, DB2, DB4, BioPatRec DB2, and UCI Gesture, respectively.	The datasets and their predefined partitions differ from a common LOSO evaluation on a low-channel wearable.
Montazerin et al. [34]	20-subject dataset, 65 gestures, 32–128 electrodes, within-subject five-fold evaluation	Compact transformer (CT-HGR)	Accuracy increased from 86.23% with 32 electrodes and 31.25 ms windows to 91.98% with 128 electrodes and 250 ms windows; instantaneous recognition reached 89.13%.	The 5.75-point increase illustrates the influence of sensor density and window duration.
Zhang et al. [41]	NinaPro DB2, NinaPro DB5, and CapgMyo DB-c	Dual-stream transformer (LST-EMG-Net)	Accuracies of 81.47%, 88.24%, and 98.95%, respectively; the dual-stream model improved average accuracy by 9.5 percentage points, with a 5.25 ms inference-time increase.	Accuracy and computational cost depend on the benchmark dataset and its partitioning protocol.
Lin et al. [45]	LOSO evaluation with one target-user calibration cycle	ConvNet with target-domain normalization	Accuracies of 87.03% and 94.53% on the evaluated 12- and 7-gesture datasets, respectively.	LOSO was calibration-assisted because normalization used a cycle from the target user.
Yang et al. [46]	LOSO on NinaPro DB2 and CapgMyo DBa; held-out subject excluded from optimization	Self-supervised pretraining and adversarial domain alignment	Calibration-free LOSO accuracies of 89.4% on DB2 and 86.9% on DBa.	Represents a stricter calibration-free cross-subject evaluation.
Peng et al. [44]	Eight subjects, four motions, intra-subject and online evaluation	Feature selection and Random Forest	Average intra-subject accuracy of 95.23% and online interactive accuracy of 95.72%.	High performance was obtained with subject-specific evaluation and should not be compared directly with LOSO accuracy.
Present study	Ten subjects, three HRC gestures, eight channels at 500 Hz, LOSO with target-specific unsupervised normalization	Factorial pipeline evaluation, ReliefF consistency analysis, and Bagged Trees	Best factorial configuration: 67.31%; five-feature baseline: 69.86%; 94.79% dimensionality reduction and 51.62% reduction in training time.	Cross-subject evaluation on a consumer wearable; target-specific normalization and global feature-count selection limit a strictly calibration-free interpretation.

**Table 2:** Sixteen sEMG Preprocessing Pipelines.

ID	Pipeline	F	O	E	N
1	R	0	0	0	0
2	R → F	1	0	0	0
3	R → O	0	1	0	0
4	R → E	0	0	1	0
5	R → N	0	0	0	1
6	R → F → O	1	1	0	0
7	R → F → E	1	0	1	0
8	R → F → N	1	0	0	1
9	R → O → E	0	1	1	0
10	R → O → N	0	1	0	1
11	R → E → N	0	0	1	1
12	R → F → O → E	1	1	1	0
13	R → F → O → N	1	1	0	1
14	R → F → E → N	1	0	1	1
15	R → O → E → N	0	1	1	1
16	R → F → O → E → N	1	1	1	1

F: Butterworth LP Filter; O: Hampel Outlier Removal; E: RMS Envelope; N: Min–Max Normalization.

**Table 3:** Time-domain sEMG features extracted per sliding window of *N* samples.

#	Feature	Formula	Description
1	Mean	$\frac{1}{N}\sum_{i=1}^{N}x_{i}$	Average amplitude of the signal.
2	SD	$\sqrt{\frac{1}{N\;-\;1}\sum_{i=1}^{N}{\left(x_{i}-\bar{x}\right)}^{2}}$	Dispersion of amplitude values around the mean or variability of muscle activation intensity.
3	VAR	$\frac{1}{N\;-\;1}\sum_{i=1}^{N}{\left(x_{i}-\bar{x}\right)}^{2}$	Squared variability of muscle activation intensity; it amplifies large deviations.
4	RMS	$\sqrt{\frac{1}{N}\sum_{i=1}^{N}{\left|x_{i}\right|}^{2}}$	Power of the sEMG, which is directly related to the level of muscle contraction force [52].
5	KURT	$\frac{N^{-1}\sum_{i=1}^{N}{\left(x_{i}-\bar{x}\right)}^{4}}{{\left[N^{-1}\sum_{i=1}^{N}{\left(x_{i}-\bar{x}\right)}^{2}\right]}^{2}}$	Quantifies the peakedness of the amplitude distribution. A high kurtosis is associated with brief, intense muscle contractions, whereas a low value suggests a more sustained and uniform activation pattern [54].
6	MMAV	$\begin{array}{cc} & \frac{1}{N}\sum_{i=1}^{N}|w_{i}\left|\right|x_{i}|\\ & w_{i}\;=\;\left\{\begin{array}{cc} \frac{4i}{N}, & i<0.25N\\ 1, & 0.25N\leq i\leq 0.75N\\ \frac{4(i-N)}{N}, & i>0.75N \end{array}\right. \end{array}$	Weighted mean absolute value that reduces the contribution of samples at the edges of the window, where muscle activation may be transitioning. It is a more reliable estimate of contraction amplitude, less sensitive to boundary artifacts [51].
7	AAC	$\frac{1}{N\;-\;1}\sum_{i=1}^{N-1}\left|x_{i+1}-x_{i}\right|$	Measures the mean rate of change between consecutive samples. It captures the speed of amplitude fluctuations [51].
8	WFL	$\sum_{i=1}^{N-1}\left|x_{i+1}-x_{i}\right|$	The cumulative length of the signal waveform over the window. It indicates a measure of the waveform related to time and amplitude, providing a measure of the signal complexity [55].
9	SSC	$\sum_{i=2}^{N-1}1\;\left[\left(x_{i}-x_{i-1}\right)\cdot \left(x_{i}-x_{i+1}\right)>0\right]$	Counts the number of local extrema in the signal by detecting changes in the sign of the slope between consecutive samples [56].
10	AE	$\frac{1}{N}\sum_{i=1}^{N}x_{i}^{2}$	It emphasizes higher-amplitude events more strongly than RMS and provides a measure of the total signal energy per sample within the window [52].
11	SKEW	$\frac{\frac{1}{N}\sum_{i=1}^{N}{\left(x_{i}-\bar{x}\right)}^{3}}{{\left[\sqrt{\frac{1}{N}\sum_{i=1}^{N}{\left(x_{i}-\bar{x}\right)}^{2}}\right]}^{3}}$	Quantifies the asymmetry of the amplitude distribution. It can differentiate activation phases where the signal is predominantly in one polarity [54].
12	CoV	$\mathrm{SD}\;/\;\bar{x}\;\left(\bar{x}\neq 0\right)$	The ratio of standard deviation to mean, expressing amplitude variability relative to the signal level [57].

**Table 4:** Mixed-effects analysis of variance (ANOVA) for testing accuracy, including effect size ($\eta_{G}^{2}$).

Source	DF	Adj SS	Adj MS	F-Value	*p*-Value	$\eta_{G}^{2}$
Pipeline	15	595,394.83	39,692.99	914.23	<0.001	0.320
Window Size	12	13,112.10	1092.68	25.17	<0.001	0.010
Overlap	8	20,392.89	2549.11	58.71	<0.001	0.016
Subject	9	532,392.54	59,154.73	1362.49	<0.001	0.296
Pipeline × Window Size	180	5182.60	28.79	0.66	1.000	0.004
Pipeline × Overlap	120	3303.59	27.53	0.63	0.990	0.003
Window Size × Overlap	96	6071.30	63.24	1.46	0.002	0.005
Pipeline × Window Size × Overlap	1440	23,285.16	16.17	0.37	1.000	0.018
Error	16,839	731,094.24	43.42			
Total	18,719	1,930,229.26				

**Table 5:** Top 10 Performance Ranking.

Rank	ID	Pipeline	Window Size	Overlap (%)	Accuracy (%)	Macro F1 (%)
1	2	R→F	650	90	67.31	67.26
2	2	R→F	500	80	66.86	66.03
3	1	R	500	90	66.77	66.08
4	8	R→F→N	500	90	66.76	66.40
5	2	R→F	450	90	66.61	66.01
6	13	R→F→O→N	650	90	66.55	65.75
7	2	R→F	750	90	66.50	66.05
8	8	R→F→N	650	90	66.47	66.22
9	8	R→F→N	700	90	66.27	65.79
10	13	R→F→O→N	450	90	66.20	65.84

**Table 6:** Mean classification accuracy and standard deviation across 10 subjects under LOSO CV for five feature-hyperparameter configurations.

Configuration	Accuracy (%)	Std. Dev. (σ)
Base All96	68.10	12.99
Base Top5	69.86	12.55
HPO All96	70.55	11.50
HPO Top5	65.50	13.22
HPO Transfer	65.97	12.21

**Table 7:** Classification performance metrics per subject for the optimal configuration.

Subject	Sex	BMI	Accuracy (%)	F1-Score (%)	Sensitivity (%)	Specificity (%)
1	M	28.73	84.31	84.24	83.91	91.15
2	M	22.77	81.21	79.84	79.77	89.96
3	M	27.38	61.38	57.89	56.55	77.05
4	F	27.93	56.38	57.79	56.78	76.63
5	M	26.70	71.90	71.55	71.03	84.44
6	F	26.35	52.59	54.57	61.49	77.09
7	M	24.00	77.07	77.53	79.08	87.93
8	F	21.34	54.83	53.80	54.14	76.05
9	F	23.44	85.00	84.85	84.37	91.46
10	F	21.30	73.97	73.88	74.71	86.32
Mean	-	24.99	69.86	69.59	70.18	83.81
Std. Dev.	-	2.76	12.55	12.42	11.93	6.47

**Table 8:** Traceability of all reported accuracy values to their configuration, feature set, and data partition, with 95% confidence intervals. All configurations were evaluated under LOSO cross-validation using the deterministic seed rng(test_subj+pipeline_id×1000); the per-repetition seed modification described in Section 4 was used only for the AAC–WFL interchangeability test and does not affect the values reported here. Confidence intervals are normal-theory intervals for the mean over the ten LOSO folds ($t_{9,\;0.975}=2.262$, $\mathrm{CI}=\bar{x}\pm t\;\sigma /\sqrt{10}$).

Configuration	Summary	Feature Set	Acc. (%)	σ	95% CI
Factorial analysis	Best preprocessing and segmentation combination from the factorial search (P2, 650 samp., 90% overlap).	Top-20 (fold-wise ReliefF)	67.31	12.46	(58.40, 76.22)
Elbow All96	Full-feature reference of the elbow curve (n=96).	Full 96	67.41	11.83	(58.95, 75.87)
Base All96	Fixed hyperparameters, full feature set.	Full 96	68.10	12.99	(58.81, 77.39)
Elbow Top5	Five features selected automatically by the consistency + ReliefF ranking.	Top-5, automatic (AAC, WFL, and SSC)	69.70	11.06	(61.79, 77.61)
Base Top5	Final model after substituting AAC with the equivalent WFL features.	Top-5, final (WFL, and SSC)	69.86	12.55	(60.88, 78.84)
HPO All96	Bayesian hyperparameter optimization on the full feature set.	Full 96	70.55	11.50	(62.32, 78.78)
HPO Top5	Bayesian hyperparameter optimization on the top-5-feature set.	Top-5	65.50	13.22	(56.04, 74.96)
HPO Transfer	HPO All96 model re-evaluated on the top-5-feature set.	Top-5 (from HPO All96)	65.97	12.21	(57.24, 74.70)

**Table 9:** Per-class precision, recall, and F1-score for the Base Top5 configuration, aggregated over the ten LOSO folds.

Class	Precision (%)	Recall (%)	F1-Score (%)
Neutral	73.24	68.90	71.00
Ask	55.93	67.66	61.24
Take	81.41	74.00	77.53
Macro mean	70.19	70.18	69.92

## Data Availability

The data presented in this study are available on request from the corresponding author due to privacy restrictions.
